# A systematic review and thematic synthesis of qualitative studies exploring GPs' and nurses' perspectives on discussing weight with patients with overweight and obesity in primary care

**DOI:** 10.1111/obr.13151

**Published:** 2020-12-06

**Authors:** William Warr, Paul Aveyard, Charlotte Albury, Brian Nicholson, Kate Tudor, Richard Hobbs, Nia Roberts, Sue Ziebland

**Affiliations:** ^1^ Nuffield Department of Primary Care Health Sciences University of Oxford Oxford UK

**Keywords:** behavioural interventions, obesity, primary care, qualitative

## Abstract

Guidelines and evidence suggest primary care clinicians should give opportunistic interventions to motivate weight loss, but these rarely occur in practice. We sought to examine why by systematically reviewing qualitative research examining general practitioners' (‘GPs’) and nurses' views of discussing weight with patients. We systematically searched English language publications (1945‐2018) to identify qualitative interview and focus group studies. Thematic methods were used to synthesise the findings from these papers. We synthesised the studies by identifying second‐order themes (explanations offered by the original researchers) and third‐order constructs (new explanations which went beyond those in the original publications). Quality assessment using the Joanna Briggs checklist was undertaken. We identified 29 studies (>601 GPs, nurses and GP trainees) reporting views on discussing weight with patients. Key second‐order themes were lack of confidence in treatments and patients' ability to make changes, stigma, interactional difficulty of discussing the topic and a belief of a wider societal responsibility needed to deal with patients with overweight and obesity. The third‐order analytical theme was that discussions about weight were not a priority, and other behavioural interventions, including those relating to smoking, often took precedent. GPs and nurses reported that noting body mass index measurements at every consultation alongside a framework to deliver interventions would likely increase the frequency and perceived efficacy of behavioural weight interventions. GPs and nurses acknowledge the importance of obesity as a health issue, but this is insufficient, particularly amongst GPs, for them to construe this as a medical problem to address with patients in consultations. Strategies to implement clinical guidelines need to make tackling obesity a clinical priority. Training to overcome interactional difficulties, regular weighing of patients and changing expectations and understanding of weight loss interventions are also probably required.

## 1 INTRODUCTION

Several national guidelines recommend that primary care physicians should identify patients with obesity and provide treatment options, including brief opportunistic behavioural interventions.^1^, ^2^, ^3^ A recent trial showed direct evidence for the effectiveness and acceptability of a primary care opportunistic^4^ intervention which offered referral to a behavioural weight‐management programme.^5^ If implemented at a population level, this could reduce the projected annual incidence of heart disease, hypertension and diabetes by 22%, 23% and 17% by 2035.^5^ Despite such evidence and guidelines, primary care weight management interventions are rare and declining. In the United Kingdom, for example, only 3% of people with obesity are referred by general practitioners (GPs) for weight loss support despite obesity prevalence being 27% and the average person visiting their GP six times per year.^6^, ^7^ Survey data have shown that weight management counselling of patients with obesity visiting their GP in US primary care declined from 33% in 2008 to 2009 to 21% in 2012 to 2013 and the reported prevalence of obesity in primary care records is considerably underestimated.^8^ In the United Kingdom, surveys suggest that a minority of patients with overweight (17%) or obesity (42%) recalled ever having been offered weight loss advice by primary care nurses and GPs.^9^ Surveys suggest that GPs believe that obesity does not belong in the medical domain,^10^ whilst qualitative evidence from interviews suggest that weight loss discussions are an inappropriate use of their time and also worry about damaging the relationship with the patient.^11^ Conversely, patients with overweight report being open to receiving GP advice on weight loss, with less than 1% describing it as inappropriate.^5^, ^12^ A systematic review on this topic^13^ uncovered largely quantitative studies, (11/13) (studies were surveys and questionnaires), describing GPs' and nurses' views, finding that weight is awkward to discuss. There have been two^14^, ^15^ exclusively qualitative reviews investigating this topic. One^14^ focused on the issue of stigma, but because of the narrow search criteria (studies *had* to mention stigma), it did not examine other barriers to conversations (such as time, lack of skills and confidence) nor did it explore clinical implications—for example, it did not examine attitudes towards guidelines. This criterion also limited the breadth and number of studies, including two studies of GPs and five studies of primary care nurses. The other qualitative review by Dewhurst et al.^15^ provided insights into GPs' reported views and experiences but missed key studies related to physicians' in training, nurses' experiences and studies related to communication with patients, perhaps because the electronic search was too narrow. We therefore aimed to provide a comprehensive overview of GPs' and nurses' views of treating obesity in primary care using broader selection criteria than either of the other published reviews. Effective strategies to implement obesity guidelines need to understand how GPs and nurses view treating obesity if they are to succeed. We undertook a systematic synthesis of research with GPs and nurses to understand (i) why conversations with patients with overweight are infrequent and (ii) to identify potential mechanisms to increase the frequency of discussions in practice.

## 2 METHODS

### 2.1 Terminology

This study included papers from many different countries with different terms for family doctors, GPs, family nurses and practice nurses. For consistency and clarity, we will call all types of family practitioners (GPs) and will call all primary care nurses, practice nurses and family nurses (nurses).

### 2.2 Search strategy

The search criteria used terms relating to (1) primary care, (2) overweight and obesity and (3) discussions and communications about weight (example of search code displayed in Box 1). During the development of the search, we trialled wider search terms, which included ‘cultural influences’ affecting the discussion of weight, but these did not yield additional studies. We also used forward and backward citation searches, which yielded two additional studies.

Box 1: **Example of search code**

As an example, the following is the code used for PubMed:

Search (((((("Health Communication"[Mesh]) OR "Professional‐Patient Relations"[Mesh])) OR (talk* OR communicat* OR conversation* OR "raise the issue" OR "raising the issue" OR "raise the topic" OR "raising the topic" OR "broach the topic" OR "broaching the topic" OR “raise the subject” OR “raising the subject” OR “broach the subject” OR “broaching the subject” OR counsel* OR advice OR advising))) AND (((overweight OR obes* OR weight^1^ OR "lose weight" OR "losing weight" OR "weight loss")) OR ((("Overweight"[Mesh] OR "Weight Loss"[Mesh:NoExp])) OR (overweight OR obes* OR weight^1^ OR "lose weight" OR "losing weight" OR "weight loss")))) AND (((("General Practice"[Mesh] OR "Office Visits"[Mesh])) OR ("General Practitioners"[Mesh] OR "Physicians, Family"[Mesh] OR "Physicians, Primary Care"[Mesh] OR "Nurses, Community Health"[Mesh])) OR (general practi* OR family practi* OR family physician* OR family doctor* OR primary care physician* OR primary care doctor* OR primary care nurse* OR practice nurse* OR community nurs* OR health visitor*)) Filters: English

### 2.3 Information sources

Searches were carried out using an emergent rather than an exhaustive strategy, following an approach used to address other complex public health questions.^16^ The reviewers identified relevant search terms, which were then further explored by an information specialist (Nia Roberts). Preliminary searches were conducted to check whether known relevant papers were identified. An initial search of the electronic databases PubMed, Embase, Cinahl, Web of Science and PsycINFO was undertaken. Searches were conducted for papers published in peer‐reviewed journals, written in English with publication date between 1945 and 8 October 2018 and supplemented with forward and backward citation searches. The information related to coverage dates of each source is detailed as follows in Box 2.

Box 2: **Sources and coverage dates**

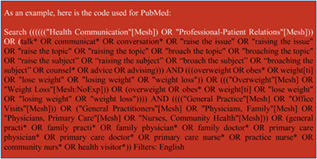

### 2.4 Study selection

We included studies which were carried out in primary care, reported the perspectives of primary care staff in discussing overweight and obesity with adults (18+ years) who were overweight and included qualitative studies based on interviews or focus groups in which GPs and nurses reported their views about discussing overweight.

We excluded studies carried out exclusively in specialist health care, studies that reported GPs' and nurses' views of discussing excess weight with children, parents or pregnant women, studies exclusively about the attitudes of patients rather than health‐care professionals, quantitative studies involving surveys or questionnaires and discussions exclusively concerning underweight or anorexia. Abstracts were excluded because they are too short for useful thematic analysis. Books and dissertations were excluded for practical reasons.

Title and abstract screening was conducted by two independent reviewers (WW and CA) using Covidence. In the event of disagreement, the article proceeded to the next phase.^17^ Full text screening was done by WW, and two 10% samples were checked by other authors.

### 2.5 Quality assessment and risk of bias

We appraised data quality using the Joanna Briggs checklist, which has been recommended as the most coherent and comprehensive tool to capture study quality.^18^ Based on the results of this checklist, we assessed whether the ‘higher‐quality’ studies contributed richer data to the thematic analysis We found that there was no such relationship and therefore included all papers.^17^

### 2.6 Methods for data extraction and a thematic synthesis

We included both the results and interpretations sections of papers, following the approach of Thomas and Harden.^19^, ^20^ Thematic synthesis involved three overlapping stages: (i) free line‐by‐line coding of the all findings and discussion sections of the primary studies using NVivo software (version 11)^21^; (ii) the consolidation of these ‘free codes’ into related areas to develop overarching ‘descriptive themes’ which were completed by organising the results into a mind‐map (WW, CA and BN)^22^; (iii) and the development of analytical themes, which directly addressed the aims of the review. In meta‐ethnography, the equivalent to this last stage of ‘analytical themes’ is ‘third‐order interpretations.’^23^

### 2.7 Protocol and registration

We wrote a protocol which was supplied to the editor of the journal.

## 3 RESULTS

**FIGURE 1:**
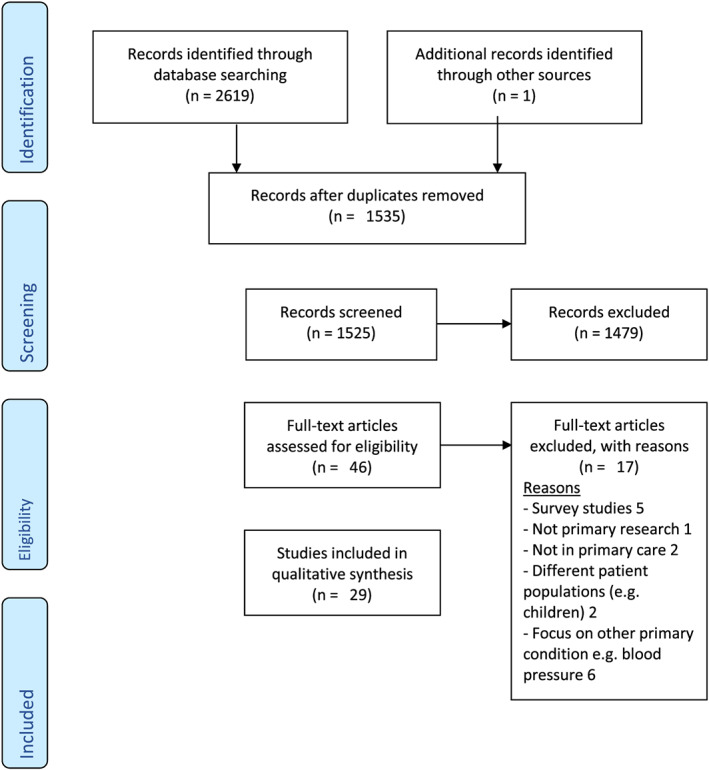
PRISMA flow chart of included studies

The search identified 1,525 nonduplicated studies, of which 29 were included for final analysis after a screening process displayed in Figure 1. Qualitative data from the included studies provided data for 601 GPs and nurses. The earliest paper was published in 2001.

### 3.1 Study quality and risk of bias

There were some quality issues. Some studies did not report ethical approval^11^, ^24^; authors drew conclusions that did not flow from the data^25^ or did not discuss reflexivity.^26^, ^27^ Recruitment to the study group was sometimes self‐selected from a subgroup with training in weight management^28^, ^29^, ^30^ or recruitment was incentivised by offering educational obesity treatment training,^27^ which perhaps meant that clinicians with greater interest were recruited.

### 3.2 Descriptive themes summary

Table 1 demonstrates the similarity of findings across different countries, between 2001 and 2017. The themes are the interactional difficulty of raising the topic in the consultation, a lack of confidence in treatments, a lack of confidence in patients to make changes, lack of knowledge and skills, insufficient time or resources, the suggestion that weight loss is not their clinical responsibility and stigma. Table 1 also illustrates that there has been little change in the number or types of themes discussed since 2001. In some instances in the following sections, descriptive themes are discussed together to save space.

**TABLE 1:** Summary table of included studies with descriptive themes from thematic synthesis

Study information				Descriptive themes						
Study name	Country	GP and/or nurse	Study type, e.g., focus groups	Lack of confidence in treatments	Lack of confidence in patients' ability to follow treatments	Awkward nature of discussing weight	Lack of knowledge/skills	Lack of resources/competition for resources	Not GPs' or nurses' responsibility	Stigma
Mercer and Tessier^11^	UK	10 GPs 10 nurses	Interviews after survey	✓	✓	✓	✓	✓		
Huang et al.^31^	US	24 GPs	Focus groups	✓	✓					
Douglas et al.^32^	SCO	10 nurses	Interviews after questionnaire				✓	✓		
Epstein and Ogden^33^	UK	21 GPs	Interviews	✓		✓	✓		✓	
Ribera et al.^24^	ESP	33 GPs and Nurses	Interviews and focus groups	✓	✓	✓	✓	✓		
Forman‐Hoffman et al.^34^	US	Seven GPs	Focus groups	✓	✓	✓		✓		
Alexander et al.^35^	US	17 GPs	Focus groups		✓	✓	✓		✓	
Brown et al.^36^	UK	15 GPs	Interviews after survey		✓	✓				✓
Leverence et al.^37^	US	14 GPs and nine nurses	Interviews	✓	✓	✓		✓	✓	
Ampt et al.^28^	AUS	15 GPs and one nurse	Interviews (after prevention trial)				✓	✓	✓	
Ali et al.^38^	UAE	15 GP and five nurse	Interviews (new sample)						✓	
Jochemsen‐van der Leeuw et al.^39^	NL	13 GP trainees and 12 trainers	Focus groups (new sample)	✓	✓	✓	✓		✓	
Hansson et al.^40^	SWE	10 GPs and 10 nurses	Interviews		✓	✓		✓	✓	✓
Heintze et al.^41^	DEU	15 GPs	Interviews		✓	✓			✓	
Derksen et al.^42^	NL	12 GPs and three nurses	Interviews and focus groups	✓		✓				
Gudzune et al.^30^	US	24 GPs and two nurses	Focus groups (after obesity trial)	✓	‘✓	✓	✓	✓		
Gunther et al.^43^	UK	Seven GPs and seven nurses	Interviews			✓	✓	✓	✓	✓
Nolan et al.^26^	UK	22 nurses	Interviews	✓	✓		✓	✓	✓	
Sonntag et al.^44^	DEU	15 GPs	Interviews		✓	✓.			✓	
Phillips et al.^45^	UK	18 nurses	Interviews	✓	✓	✓	✓			
Claridge et al.^46^	NZ	12 GPs	Interviews	✓		✓			✓	✓
Blackburn et al.^47^	UK	17 GPs and 17 nurses	Interviews			✓	✓	✓		✓
Kim et al.^48^	AUS	12 GPs	Interviews	✓	✓	✓				
Teixeira et al.^4^	PRT	16 GPs	Interviews	✓	✓				✓	
Ashman et al.^29^	AUS	12 GPs	Interviews	✓		✓	✓			
Antognoli et al.^27^	US	Nine GPs	Interviews with most knowledgeable GP trainers after survey		✓	✓	✓	✓	✓	✓
Asselin et al.^49^	CAN	15 nurses	Interviews (participants from obesity trial)						✓	
Glenister et al.^50^	AUS	Seven GPs and seven patients	Interviews	✓		✓	✓	✓	✓	✓
Lee et al.^25^	SGP	50 GPs	Interviews and focus groups	✓		✓				
Total				17	17	22	15	13	16	7

Abbreviation: GP, general practitioner.

### 3.3 Descriptive themes

#### 3.3.1 The awkward nature of weight discussions

The interactional delicacy of the topics was a key theme addressed in 22 of 29 papers. It covered a number of subthemes described as follows.

##### Word choice

Clinicians reported word choice as a barrier, with concern particularly that their patients might be offended and rapport damaged if terms such as ‘overweight’ or ‘obesity’ were used.^25^, ^27^, ^36^, ^44^, ^45^, ^47^, ^48^, ^50^

This experience was similar across many different countries in both public and private settings: a UK GP reported that using these terms was not ‘very PC (politically correct)’ and could cause patients to get ‘very hurt’^47^; another Singapore GP in a private practice reported patients being offended by such terms.^25^

It's a very sensitive subject you cannot tell the patient ‘Oh by the way, I think you are obese’ because you'll end up offending them, they'll never come to your clinic again [laughs]. GP, Singapore (who ran several private clinics)^25^

Clinicians reported softening of terms and generally avoiding the term obesity because of its negative connotations.^51^ Instead clinicians reported asking more general questions about whether the patient had ever tried to lose weight. The word choice at the start of the conversation when broaching the topic was deemed most difficult.

I find it quite difficult because I do not want to offend someone, I do not wanna start off by putting them on sort of the back foot. Nurse UK^51^

##### Difficulty making progress on a complex problem in time constraints

Weight and obesity were reported as too complex to deal with in a 10‐min appointment,^50^ especially if a patient was presenting for another reason.^27^, ^34^, ^47^ The complexity of the subject was attributed to the belief that obesity was related to many other aspects of a patient's life^46^, ^50^ and that behaviour change was a long‐term process requiring long‐term management.^42^ The personal and societal roots of the issue made clinicians feel disempowered to properly address within the time constrains of their consultations.^46^

##### Comparative difficulty of assessing obesity

Some GPs, nurses and GP trainees suggested that obesity was more difficult to discuss than smoking^39^ because smoking was seen as a more accepted risk factor^47^ and more straightforward to assess^28^ and treat.^39^ There was a perception that smoking was a clear choice in behaviour whereas obesity was a consequence of a long‐term aggregation of several behaviours.

Smoking is more a choice while becoming overweight just happens. GP, New Zealand^46^

These behaviours (such as diet and physical activity) were often inferred by appearance, only being assessed if the patient was visibly overweight.^28^

##### Reported mechanisms used to broach the awkward topic

A long‐term trusting relationship between the patient and clinician^30^, ^39^, ^40^, ^41^, ^45^, ^50^ eased discussion. Clinicians also found that objective ‘medicalised’ body mass index (BMI) measurements, or reading guidance from the computer, helped them feel more comfortable.^25^, ^30^, ^33^, ^37^, ^40^, ^46^, ^51^

I actually, sort of, put it sideways and say, ‘Well the computer's saying that, in fact, you are overweight or obese’ Nurse, UK^51^

Clinicians described linking discussions of weight to relevant medical concerns.^25^, ^30^, ^33^, ^37^, ^40^, ^46^, ^51^ The topic was also seen as less interactionally difficult when they positioned the issue as more ‘doctorable’ (e.g., if there was severe obesity^28^, ^33^ and bariatric surgery^48^ was a treatment option.

For those seriously overweight … who are either encountering medical problems, or at a high risk of medical problems, and actually probably what we should be taking is a more medical medicalised approach. UK, GP^33^

Some GPs and nurses said they felt more comfortable reframing weight loss discussions as recommendations about maintaining health.^30^, ^51^ GPs and nurses also speculated that if they were ‘forced’ to intervene by some mechanism, they would be more likely to discuss weight.^28^

#### 3.3.2 Lack of confidence in treatments and patients' ability to follow treatments, and lack of knowledge and skills

Three themes widely developed across studies: a lack of confidence in the treatments (reported in 17 studies), clinicians' lack of knowledge and skills to support weight loss (cited in 15 studies) and lack of confidence in patients' abilities to make changes and sustain weight loss (cited in 17 studies).

##### Lack of confidence in treatment

Clinicians' lack of confidence in treatment was a key theme addressed in 17 of 29 papers. It covered a number of subthemes described as follows.

##### Lack of previous success

Past experience of treatments' lack of success often made clinicians doubtful about offering them,

I just have not seen it be very successful with very many people. … I mean the reality is [that] you know from everywhere you look weight loss does not work very well for most people. GP, USA^37^

Clinicians also worried about recommending a treatment they had little faith in because this could damage their credibility with the patient.^33^

##### Lack of confidence in skills

Lack of specialist skills to talk about what was believed to be a very complex topic gave physicians reservations about broaching the subject,

I do not feel confident to really get into the nitty gritty of… patients' questions about this diet, this food and that food, and I think, oh, I'll leave that to somebody else to do. GP Australia^29^

The lack of confidence in skills was in some instances blamed on lack of a standardised approach to raising the issue.^47^ Despite this, some clinicians attempted interventions drawing from personal experience and media sources.^47^

##### Lack of confidence in patients' ability to make changes

Clinicians doubted that patients had the ability to make changes with some suggesting that the prevalence of obesity in society was proof that individuals' weight loss strategies did not work.^37^ They expressed these thoughts sometimes in pejorative terms.

You can lead a horse to water but you cannot stop it eating cream cakes. GP, UK^43^

This pessimism was sometimes presented as borne of experience, but some acknowledged their own lack of skills^29^ and knowledge of effective treatment,^37^ which contributed to a reported feeling of powerlessness.^33^ Some GPs said that they wanted to encourage patients to lose weight but did not know how to do so,^29^ whilst others spoke pejoratively about patients who were reluctant to change.^46^

##### Lack of confidence in existing guidelines and metrics of success

Some GPs believed that following guidelines which encouraged clinicians to use weight metrics and to judge success by weight or BMI ‘as yardsticks of success’ was the wrong approach. Furthermore, this damaged their self‐efficacy since they knew they did not believe what they were being told to recommend and track would help people lose weight.

I do not want to be falsely saying… ‘I really believe if you do this this would be effective’… GP Australia^29^

Authenticity was also felt more challenging by those clinicians with a lower personal BMI.^51^

National guidelines were judged by some as needing to be localised, taking into account local needs and variances in obesity service provision.^43^ Others were reluctant to follow national guidelines,^35^ preferring to draw on and adapt personal experience about what techniques had been successful interventions.^45^

##### Mechanisms to improve clinicians' confidence in prospect of change

Across several studies, clinicians reported that tracking patients' weight had made them less pessimistic.^25^, ^30^, ^33^, ^37^, ^40^, ^46^, ^51^ Even where this system was not in place, some GPs reflected that they thought it would help.^29^

Gudzune describes clinicians' belief that by acknowledging any degree of weight loss success provided positive reinforcement.

‘You've lost six pounds since you were here last.’ [Patients] really need that positive feedback that we are paying attention to what they are doing. GP, Singapore^30^

Nurses in a UK study said they found it effective to link weight loss to a future social event in patients' lives (e.g., wedding) to incentivise change.^45^ Similarly linking patients' weight to wider objective health measurements was perceived to keep patients motivated.^30^ Another approach was to moderate GPs' and nurses' expectations so that modest weight loss, or no weight gain, could be seen as an achievement. This approach of encouraging either moderate weight loss or preventing weight gain was observed to ameliorate GPs' sense of frustration.^11^, ^29^

#### 3.3.3 Responsibility

Another highly developed descriptive theme, reported in 20 studies, was that it was not GPs' and nurses' responsibility to intervene. These papers discussed subthemes, including the boundaries of medical responsibility, patients' responsibility, differences in role perceptions between GP and nurses and the role of the clinician in society.

##### Medical responsibility does not include treating obesity unless severe

There was concern about medicalising what many viewed as a nonmedical problem^4^, ^26^, ^42^, ^47^ for which society, the patient or the family should take responsibility.^35^

I do not think you should take it for granted that we are the ones to intervene. We're trained in medical care. Overweight and obesity are more of a societal problem. GP, Sweden^40^

However, if obesity was deemed to be ‘severe’ (sometimes^45^ defined by clinicians as BMI ≥ 35), clinicians did acknowledge the necessity to intervene.^4^, ^33^, ^45^

##### Patients' responsibility

GP participants in a study exploring the delivery of a lifestyle behavioural risk factor screening and management health check often discussed a patient's motivation as if it was immoveable.^28^ Some said that once the patient had been educated regarding risk factors, the responsibility whether to act lay with the patient.^28^ In studies examining GP training, GP trainers and trainees noted that how discussing obesity with patients or in general was not prioritised in the curriculum.^27^, ^39^

##### Different responsibility between GPs and nurses

There were differences between nurses and GPs attitudes towards discussing weight. Some GPs suggested that dealing with overweight and obesity was an inappropriate use of their time or that responsibility for this task should be shifted to nurses.^11^, ^40^ Both nurses and GPs reported that talking about weight could damage their patient relationship, but nevertheless, nurses reported feeling responsible for raising the topic.^47^ No studies reported nurses saying that treating patients with obesity was not their responsibility (0/10), whereas GPs in 11/20 studies did report this.

We are not the friendly neighbour, we are health care professionals. I do see it as my professional task to tell patients about the risks of their weight …. Nurse, Netherlands^42^

##### Society's responsibility

Some GPs believed that their responsibility to intervene was somewhat undermined by a belief that the bigger actions lay with society.^35^ Some nurses said that causes of obesity, including childbirth and media influences, were too complex to resolve through a general discussion and therefore focused on patients taking greater personal responsibility. As one author^51^ summarises,

It was evident that this was a difficult course to steer, and eventually some participants would return to the importance of personal lifestyle in obesity: ‘So it is lifestyle, it is, you know, them’ Nurse UK.

Some^35^, ^37^ saw the family as responsible for a family member's weight.

‘If the family was not onboard, it would be “very hard, if not nearly impossible” to achieve a weight loss.’ GP, US.^35^

##### Contexts where GPs and nurses took responsibility for weight loss discussions

Participants in several studies reportedly felt responsible to intervene.^30^, ^35^, ^37^, ^41^, ^42^, ^46^ These examples were from the Netherlands, the United States of America, New Zealand Sweden and the United Kingdom which all have different health systems, with different payment models and consultation lengths. (Consultation lengths in the United States and New Zealand are 3‐5 min longer than in the United Kingdom or the Netherlands^52^, ^53^). If the GP saw their role as treating disease, rather than attending to risk factors, then obesity in the absence of an associated health problem was out of their sphere^27^ as this author notes ‘[Staff] considered that their main task was to treat diseases, and overweight and obesity were seen more as conditions that might involve a risk of diabetes or some other disease.’^40^

However, if a disease associated with obesity was present, then intervention could be justified as it ‘lifted the negativity and ambiguity that existed about managing obesity,’^11^ though even in such a scenario, one GP reported that it would still be a task better suited to a nurse.^40^

A final mechanism which GPs reported making them feel responsible was having a system of long‐term follow up in order to achieve sustained weight loss as this Dutch GP suggests,

There is a neat reporting system, but after the last treatment no one feels responsible.^42^

#### 3.3.4 Lack of resources and competition for resources

Doctors and nurses reported that they did not have sufficient time to address overweight^40^ and that other activities took priority.^47^

That's the trouble is not it, it's the conflict of time for all the other things that we are supposed to do in a ten‐minute consultation, of which probably smoking cessation comes quite high on the sort of health promotion thing … and alcohol, of course, that's another. Nurse, UK^47^

##### Contexts when time and resources were allocated to obesity

When there was a formalised framework, GPs and nurses reported feeling a sense of duty to make weight interventions.^4^, ^24^, ^28^ These frameworks were reported to have positively affected nurses' role adequacy and legitimacy^26^ and helped make practitioners feel supported.^43^

It is part of our functions and our workload … to engage a preventative approach, advising patients to lose weight or do … Measuring weight is now a mandatory procedure for all of us in all consultations. We do it on a daily basis for every patient, regardless if he or she is overweight or not. GP, Portugal^4^

#### 3.3.5 Stigma

Stigma was a descriptive theme that was apparent in the tone of many clinicians' responses to statements and cited by many of the primary authors.^27^, ^40^, ^41^, ^43^, ^47^, ^50^, ^51^ It can be seen in a number of themes.

##### Difficulty negotiating the stigma when advocating weight loss

Clinicians reported a worry that patients would think they were stigmatising them by talking about weight and imply they were lazy or greedy,^46^ which sometimes led them to avoid the topic.^50^ The stigmatised nature of obesity in society more generally made some clinicians avoid the topic even if they themselves did not report a stigmatised attitude towards patients.^36^ Other clinicians noted the associated psychological problems with obesity as stigmatising and sometimes assumed they had associated conditions which made conversations difficult.

Depending on their mental health, so if somebody's a bit um, if they are fragile you certainly would not be bringing up about their weight. GP, Australia^50^

##### A stigmatised attitude towards patients with obesity, especially those from poorer or certain ethnic backgrounds

Others had a stigmatised attitude towards those with obesity, describing patients as lazy and lacking in energy or indifferent to their situation.^40^ Even those who were motivated to lose weight were thought to be reluctant to make the necessary changes. And those who did seek help did so for the wrong reasons, such as wanting to wear smaller clothes.

Patients want to lose weight but they do not want to change. Start walking instead of taking the bus, and eat less, that's all there is to it. Or the motivation might be there but they do not really want to do it, only if they think it's important. GP, Sweden.^40^

This stigmatised attitude towards patients was especially apparent in papers which focused on patients on low incomes^37^ or those from certain ethnic backgrounds,^40^ with these groups reported to be less frequently counselled.

##### Contexts when stigma was overcome

Some clinicians believed that the stigma could be overcome by focusing on health‐focused approaches rather than purely weight focused approaches.^29^ These included the ‘Health at Every Size’ movement which aims to reduce weight‐related stigma by focussing on other potential health‐related benefits of interventions, by focusing more on medical endpoints such as fasting glucose or blood pressure, aiming for a maintenance of current weight (over or otherwise) or aiming for lifestyle changes such as increased physical activity which have recognised health benefits independent of weight.^29^ Others believed that stigma could be reduced by not using terms such as ‘obese’ or talking around the topic.

I just talk in terms, you know, ‘Have you ever thought, you know, trying to lose weight?’ or this sort of thing, not just saying, ‘You're obese.’ I think that they must know they are overweight—you do not want to rub it in. Nurse, UK^36^

### 3.4 Analytical theme summary

**FIGURE 2:**
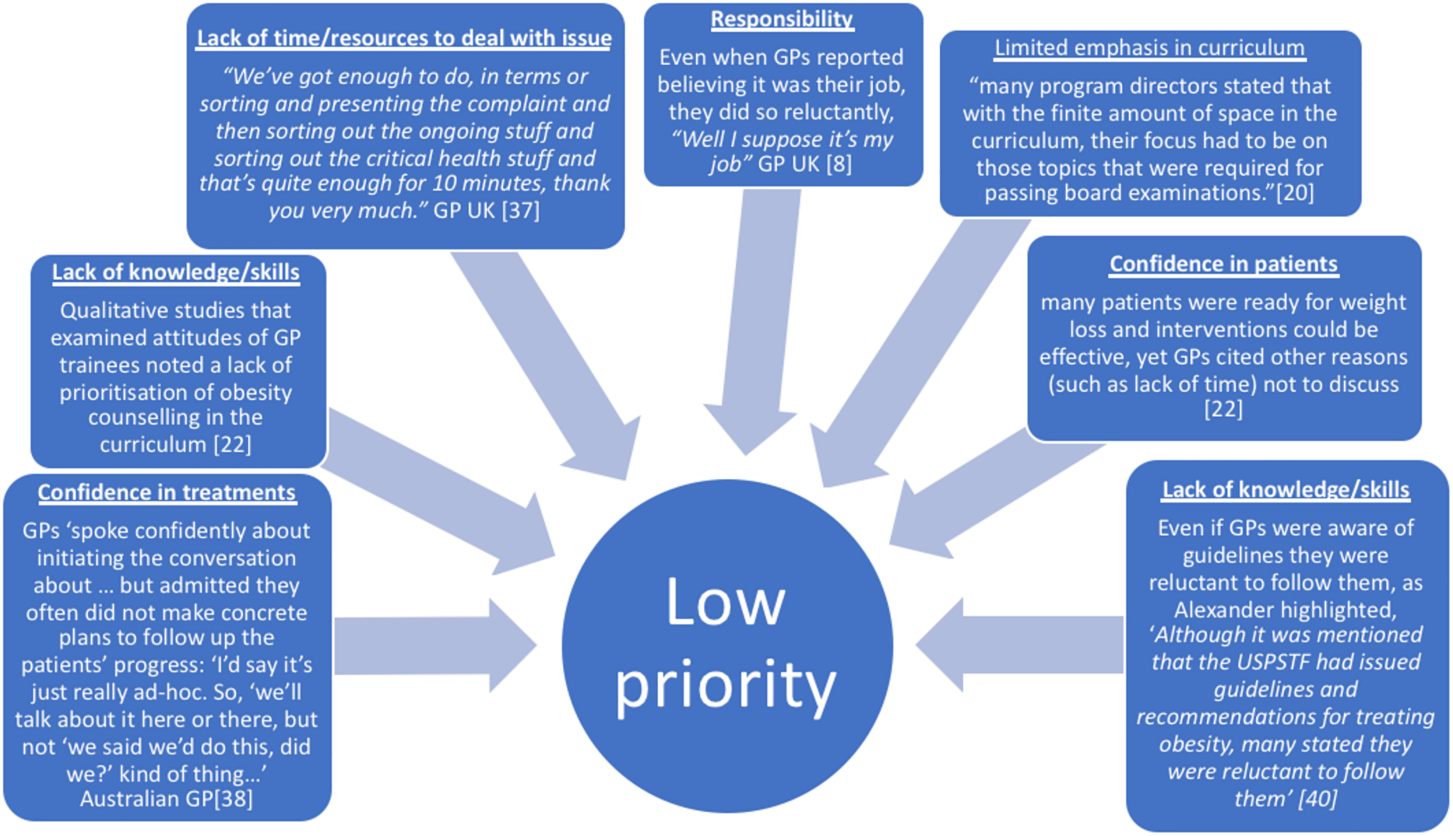
Themes contributing to the lower priority afforded to weight management discussions

Although the papers reported different clinical perspectives across different settings, there were clear similarities connecting clinicians' views towards discussing weight (Figure 2). Each of these themes contributed to clinicians affording low priority to intervening with patients on obesity. Clinicians often spoke about patients with obesity in a way that reflected society's underlying stigma about obesity.

### 3.5 Analytical theme: Low priority of weight discussions

The main overarching theme that binds the first‐ and second‐order themes was the low priority given to obesity interventions. This was remarkably consistent across studies over decades of research. This sense of low priority was shared by doctors, nurses, doctors in training and across different health‐care systems (both public and private).

#### 3.5.1 Low priority of weight discussions

Many of the descriptive themes listed in Table 1 can be viewed through the lens of priorities (Figure 2). Discussing weight was *not a priority* for GPs and nurses at all levels of seniority. This higher‐order latent theme was present even amongst those GPs and nurses who said they had confidence in their patients and in the treatments. Even those who saw discussing weight as their responsibility often did it reluctantly.^26^, ^40^

Prevention activities perceived as a second division of optional approaches … the doctor in general is more concerned with whether the patient smokes or has high cholesterol … Spanish study of GPs and nurses^24^

##### Stigma

Stigma fed into the low‐priority theme. In some clinicians' responses, their tone hinted that recipients of their care are unworthy or make themselves unworthy of their time and constructed obesity as an individual behavioural problem.^33^ Obesity was constructed by some clinicians as both a fault of individuals' behaviours whilst also noting that it was a social problem for wider society to deal with rather than be medicalised in their clinic^33^, ^34^, ^37^, ^40^ (Tables 2 and 3).

**TABLE 2:** Summary table for quality assessment using the Joanna Briggs checklist

Paper congruity	Alexander et al.^35^	Ali et al.^38^	Ampt et al.^28^	Antognoli et al.^27^	Ashman et al.^54^	Asselin et al.^49^	Blackburn et al.^47^	Brown et al.^36^	Claridge et al.^46^	Derksen et al.^42^	Douglas et al.^32^	Epstein et al.^33^	Glenister et al.^50^	Gudzune et al.^30^	Gunther et al.^43^	Hansson et al.^40^	Forman‐Hoffman et al.^34^	Heintz et al.^41^	Huang et al.^31^	Jochemsen et al.^39^	Kim et al.^48^	Leverence et al.^37^
Theoretical framework	+	+	+	−	+	+	+	+	+	+	−	+	+	−	+	+	−	+	+	−	+	+
Appropriateness of research design	+	+	‐	+	+	+	+	+	+	+	+	+	+	+	+	+	+	+	+	+	+	+
Data collection	+	+	+	+	+	+	+	+	+	+	+	−	+	+	+	+	+	+	+	+	+	+
Data analysis	+	+	+	+	+	+	+	+	+	+	−	+	+	+	+	+	+	+	+	+	+	+
Findings	+	+	+	+	+	+	+	+	+	+	+	+	+	+	+	+	+	+	+	+	+	+
Context	+	+	+	+	+	−	+	+	−	−	−	+	+	‐	+	+	−	+	+	+	+	+
Impact of investigator	‐	−	‐	−	+	−	‐	−	‐	‐	−	‐	+	−	+	+	‐	−	−	−	+	−
Believability	+	+	‐	‐	+	+	+	+	+	−	+	+	+	+	+	+	+	+	+	+	+	+
Ethics	+	+	+	+	+	+	+	+	+	−	+	+	+	−	+	+	−	+	−	−	+	−
Evaluation/outcome	+	+	+	+	+	+	+	+	+	+	+	+	+	+	+	+	+	+	+	+	+	+

Paper congruity	Lee et al.^25^	Mercer and Tessier^11^	Nolan et al.^26^	Phillips et al.^45^	Ribera et al.^24^	Sonntag et al.^44^	Teixeira et al.^4^
Theoretical framework	+	+	+	+	+	+	+
Appropriateness of research design	−	+	+	+	+	+	+
Data collection	+	+	+	+	+	+	+
Data analysis	+	+	+	+	+	+	+
Findings	+	+	+	+	+	+	+
Context	+	+	+	+	+	+	+
Impact of investigator	−	−	+	−	−	−	+
Believability	−	+	+	‐	+	+	+
Ethics	+	−	+	+	−	+	+
Evaluation/outcome	−	+	+	+	+	+	+

**TABLE 3:** Study characteristics

Author, year	Sample size	% (*f*)	% nurses	Country	BMI	Methodological and theoretical approach	Method/places of recruitment	Age range
Alexander, 2006	17	65	0	US	18 to 33 (mean 23.7; SD, 4.0)	Grounded theory	Emailed colleagues in medical Centre	29–61
Ali, 2009	29	100	34	UAE	Not given	Grounded theory	Not given.	Not given
Ampt, 2009	15	54	0	AUS	Not given	The theory of planned behaviour	Through involvement in recent similar study.	Not given
Antognoli, 2017	38	Not given	0	US	Not given	Not given	Letter (but incentivised through training for obesity and nutrition counselling)	Not given
Ashman, 2015	12	66	0	AUS	Not given	Social cognitive theory	Snowball recruitment of GPs who had agreed to deliver a pilot of an obesity management programme.	31–60
Asselin, 2016	29	97	100	CAN	Not given	Theoretical domains framework	Family practices who were randomised to the intervention of an obesity management tool.	26–68
Blackburn, 2015	34	82% combined (64% GPs 100% nurses)	50	UK	18–30 + (52% healthy weight, 32% overweight, 11% obese, 2% not specified).	Theoretical domains framework	Email to GP surgeries, thereafter snowball recruitment, practice level reimbursement for their time.	30–49
Brown, 2007	15	100	100	UK	40% healthy weight, 33% obese, 26% healthy range	Pragmatic qualitative methodology	Postal survey response about obesity management	28–57
Claridge, 2014	12	42	0	NZ	Not given	Inductive thematic analysis	33% existing connection, 67% random calling of GP clinics.	31–60+
Derksen, 2012	11	66	72	NL	Not detailed, only given in vague language e.g. ‘about one‐third seemed to be overweight’	Not detailed	Members of research team asked their colleagues.	Not detailed.
Douglas, 2005	10	100	100	SCO	Not given	Not detailed other than ‘mixed methods approach’	Recruited by virtue of them returning a questionnaire	Not given
Epstein, 2005	21	52	0	UK	Not given	Interpretative phenomenological approach	All GPs in one trust were invited but not detailed by what method.	30–60+
Glenister, 2017	7	Not given	0	AUS	Not given	Thematic analysis	Email sent to two general practice in two towns.	Not given
Gudzune, 2012	26	58	8	US	Not given	Not detailed	Recruited as a substudy from an obesity reduction trial, only practitioners who had enrolled 4 or more patients in the trial were eligible.	Mean (SD) 46.4 (10.7)
Gunther, 2012	14	85.5 combined (43% female amongst GPs, 100% amongst nurses)	100	UK	Not given	Interviews used but no theory detailed	‘Obesity leads' in trusts were aksed to identify practices with different levels of obesity	31–64
Hansson, 2011	20	65	50	SWE	Not given.	Phenomenographic	Mail/phone to medical heads of primary care centres in well‐defined area in Sweden who then referred staff.	34–60
Hoffman, 2006	6	Not given	Not given	US	Not given	Focus groups but theory not mentioned	Not detailed	Not detailed
Heintz 2011	15	60	0	DEU	None had elevated BMI (mean 22.4)	Free associated narrative method, MAYRING's qualitative content analysis and theoretical sampling	Written letter sent to 70 GPs who which 15 participated in the study	Mean age 51
Huang, 2004	24	22	0	US	Not given	Focus groups with scripted probes and encouraged participants to clarify answers	Not detailed but participants compensated between $50–100 depending on seniority	27–52
Jochemsen, 2011	25	60	Unclear because GP teachers are both GP's and behavioural scientists	NL	Not given	Focus groups with probing questions, no theory detailed	Prospective sampling by selecting every third name on an alphabetical list from GP training Centre	29–36 for trainees, trainers not detailed
Kim, 2015	24	54	0	AUS	Not given	Semistructured interviews, not wider theory detailed	Invited via email sent by primary care organisations' local liaison officers.	Not given in full detail
Leverence, 2007	23 (paediatricians not included)	43 total (but not detailed when paediatricians removed)	39	US	Not given	In depth semistructured interviews and focus groups designed to elicit encounter‐based narratives	Not detailed other than a researcher directly contacted prospective interviewers in sample taken from research in outpatient settings network	Not given
Lee, 2017	50	42	0	SGP	Not given	Grounded theory. Focus groups and in‐depth interviews.	Email and telephone contact, but no further detail	25–56
Nolan, 2012	22	95	100	UK	Not given	Semistructured face‐to‐face interviews. Interviewer worked locally for primacy care trust as an obesity lead and had organised local obesity training, and some participants were aware of this.	Invited by letter to nurses, contacted subsequently via telephone. During recruitment, potential participants were told study was about take‐up and use of NICE guidance on obesity.	Not detailed
Phillips, 2013	18	100	100	UK	Not given	F2F interviews with a thematic analysis of results.	Via email sent to lead nurses for local health board who were asked to send to all practice nurses in their area. Interested nurses contacted the research team via email or phone. 78% declared specific interest in obesity management	Not detailed
Ribera, 2005	33	Not detailed for focus groups	45	ESP	Not given	Semistructured interviews corroborated data and focus groups provided ‘in‐depth personalised information’. Stages of change theory and decisional balance concept was used. Theoretical sampling strategy was used.	Not detailed for focus groups, but overall sample was selected from seven regions of Catalan health system, it does not detail how they were contacted.	Not detailed
Sonntag, 2011	15	60	0	DEU	None had elevated BMI (mean 22.4 kg/m^2^)	Semistructured interviews with open‐ended questions, transcribed and subjected to Mayring's technique for qualitative analysis.	GPs were contacted by the local board of health in Berlin and not incentivised.	Average age 51
Teixeira, 2014	16	56	0	PRT	Average BMI 25.55/m^2^ (range 20.83–30.48 kg/m^2^).	Semistructure face to face interviews with an inductive thematic analysis of results.	GPs invited via telephone and/or after approval of heads of GP centres. After first contact, snowball sampling employed	32–57

Abbreviations: BMI, body mass index; GP, general practitioner.

I do not think you should take it for granted that we are the ones to intervene. We're trained in medical care. Overweight and obesity are more of a societal problem. GP, UK^40^

There's also the other side of what that patient is doing, or not doing, … so there's just so far that we as providers can go. You can educate but then it's on the other side, in the other party's hands. GP, America^37^

##### Financial incentives

Financial incentives were reported as making weight management a higher priority.

With the introduction of QoF, we are increasingly aware of certain things we need to address … obesity interacts with other co‐morbidities … So, you cannot ignore it. GP UK^43^

## 4 DISCUSSION

### 4.1 Statement of principal findings

Discussing weight was found consistently as not a priority for primary care GPs, GP trainees or nurses. This consistency demonstrates the imperative for policymakers to take action to improve the implementation and perceived value in obesity guidelines. The reasons clinicians gave for not intervening on obesity were as follows: that discussing weight was interactionally difficult; it was not seen as the GPs' responsibility to treat people with obesity, although nurses felt more that this was their responsibility; nurses and GPs lacked knowledge about interventions and it had not been prioritised in the GPs' curriculum; a lack of confidence in patients' ability to make changes; and a lack of confidence in the efficacy of treatments. Clinicians reflected stigmatised views of people with obesity.

### 4.2 Strengths and weaknesses of the study

A strength of this study was the qualitative thematic synthesis method, which was chosen because it has been used successfully to address questions relating to intervention need, appropriateness and acceptability.^55^ It draws on relevant elements of grounded theory (taking an inductive approach using constant comparison) and meta‐ethnography (using third order interpretations). Another strength was its focus on primary care. Brief opportunistic interventions are arguably more relevant in primary care than in other contexts,^1^ particularly in view of the long‐term regular contact,^56^ which is important given individuals' difficulties in sustaining long‐term weight loss and the impact of trust and ongoing relationships in enabling clinicians to raise this topic.

Our review was limited by the quality of available of studies as well as the quality assessment tool. The Joanna Briggs checklist was used in accordance with good practice, but there was a mismatch between the richness of the data in the studies and the score they received in the checklist, which raises questions of the usefulness of such checklists. Additionally, every study in this review asked clinicians directly about why they do not intervene to help patients who are overweight. Studies repeatedly elicited responses such as lack of time or that it is awkward to discuss. This approach is likely to be subject to social desirability bias,^57^ which encourages interviewees to present a justification for their lack of engagement with the official standard of care. Investigators appeared not to have gone beyond reporting these reasons to probe more deeply to uncover the cause of this reluctance.

### 4.3 Findings in relation to the existing literature

We identified four existing reviews in this domain, two of which were either exclusively^58^ quantitative or predominantly quantitative studies.^13^ Regarding the two remaining qualitative reviews, one^14^ was useful to contextualise health‐care workers' thoughts on obesity stigma, but it did not explore the relevant clinical implications—for example, it did not examine attitudes towards obesity guidelines. Moreover, the breadth of this previous review was limited: it only included two studies with GPs and five studies with nurses, whereas we included 23 with GPs and seven with nurses. The other qualitative review by^15^ provided insights into GPs' reported views and experiences but did not include studies related to physicians' in training, nurses' experiences and studies related to communication with patients perhaps because the electronic search was too narrow. Although the review of Dewhurst et al. included 16 studies, we included 14 of the same studies and an additional 15. By using different search terms, our review uncovered further studies, which gave a unique insight. For example, Ashman et al.^29^ explored GPs' self‐efficacy towards obesity treatments and discovered that GPs did not feel ‘authentic’ giving advice related to BMI targets, which they felt were unlikely to work, and instead reported preferring to give interventions related to positive lifestyle changes, such as exercise which may have health benefits independent of weight.

Stigma was a theme that was raised in a number of different guises by clinicians in our study, building on a wide body of work on this topic.^59^, ^60^ There was an apparent contradiction in clinicians' attitudes who could simultaneously think it was both individual's responsibility to lose weight but also caused by a wider social problems, and both these perceived causes meant that intervening was not worth the clinicians time. This mindset has been previously highlighted in analysis of moral discourse in clinicians accounts about weight counselling,^61^ which they noted contributed to a feeling of both tension and powerlessness amongst clinicians. Our study built on this individual versus society analysis and found that this attitude became more acute towards patients from poorer backgrounds, who they deemed lazier,^37^ and towards groups from certain ethnic backgrounds, whose cultures did not prioritise weight loss interventions.^46^

We also built on existing literature that had highlighted that clinicians may not always stigmatise individuals but find it hard to negotiate stigma when advocating weight loss.^61^ Our study also complemented literature^62^ which noted the impact of weight stigma on patients' care, finding that experiences of perceived substandard care may cause avoidance of care, poor adherence to weight management interventions and distrust of clinicians. This was reflected in our own findings of perceived physicians' frustrations in patients' adherence with interventions.

### 4.4 Meaning of the study: Possible mechanisms and implications for GPs, nurses and policymakers

The findings suggest that GPs see intervening on obesity as a low priority. This feeling was not so marked for nurses. The lack of confidence in the patients and treatments sometimes gave the impression that it was not that GPs did not *have* sufficient time, but treating obesity was not *worth* their time [1], sometimes related to an underlying stigma towards those with obesity.^50^ What little time they did have should be used to treat priorities such as disease and more important prevention activities.^24^ It appears therefore that GPs do not want to intervene or do not like intervening, and some of the reasons proffered may mask this underlying sense of dislike. Why might this be so? Anthropological studies of medical training show the emphasis placed on diseases, technical procedures and technological medicine and less on the behavioural aspects of medicine, such as prevention especially areas such as obesity where there are no easily prescribed medicines.^63^, ^64^ GPs in training believe that disciplines of medicine that involve highly technical procedures or experiments, such as surgery or laboratory medicine, are more prestigious than primary care.^65^ Normalisation Process Theory proposes that interventions are adopted if clinicians value them.^66^ Nursing places less emphasis on the technological aspects, and thus nurses seem to feel more duty to act on what both groups consider to be an important risk factor for ill health. Thus, interventions to support physicians need to grapple with this cultural block and find a way to make clinicians, particularly doctors, feel that intervening on obesity is valuable work. In addition, clinicians were highly sceptical of their ability to intervene for patients and patients' ability to respond, but such beliefs can be countered with evidence, but they also need the skills and confidence to enact brief interventions. When GPs did decide to intervene, they did so in a way which evidence has shown to be unhelpful. To navigate the perceived interactionally difficult and stigmatised topic, GPs and nurses talked around the issue^50^ or linked weight to a corresponding health issue, believing they were on ‘safer ground’ and this would cause less offence to a patient.^46^, ^50^, ^67^ However, there is evidence that GPs' and nurses' attempts to link discussions of weight to the patients' own health issues can result in resistance.^56^ Other previous studies have noted the interactional delicacy and suggested that training is needed to make clinicians far more confident in navigating this delicacy.^68^

### 4.5 Summary and future perspectives

Clinicians offer a variety of explanations as to why they do not offer support to their patients to lose weight, despite national guidelines that urge them to do so. These relate to the awkward and potentially stigmatising nature of the conversation, the lack of faith that patients will change and their own lack of a clear intervention to offer. However, underlying all this is a sense that such conversations are not valued, especially by doctors, and that this relates to unspoken value systems in medicine that prize technological fixes over behavioural interventions. Addressing nonadherence with guidelines will require attention to these underlying values.

**SUMMARY BOX OF REPORTED MECHANISMS COVERED IN TEXT THAT ENABLED WEIGHT CONVERSATIONS**

1. When GPs and nurses allocate their time and resources to dealing with obesity.

- When weight measurement becomes mandatory.
- When there is a formalised template/framework.

- Reported mechanisms to address GPs' and nurses' perceived feeling of lack of responsibility.
  - Create a system of permanent follow‐up.
  - Raising awareness about obesity associated diseases.
  - Emphasise how interventions are part of a broader societal effort to tackle the multiple and complex causes of obesity.
- Reported mechanisms to improve GPs' and nurses' confidence in patients and treatments:
  - Linking weight loss to an upcoming event;
  - Seeking wider objective health changes, like blood pressure, to keep patients motivated;
  - Having a system of follow up.
  - Moderate GPs' and nurses' expectations to seek only modest weight loss or no weight gain.
- Reported contexts and mechanisms to make discussing weight less interactionally difficult:
  - Using computer prompts.
  - Routinely recording the patients' BMI.
  - Linking the patient's weight to comorbidities and medicalizable conditions.*
  - Having a long‐term trusting relationship.
  - When there is severe obesity and the issue becomes more doctorable, particularly if bariatric surgery is a treatment option.
  - Referring to broader ‘health’ changes rather than ‘weight’ changes.

## CONFLICTS OF INTEREST

PA obtained funding from Cambridge Weight Plan for an investigator‐initiated clinical trial. PA spoke at a symposium at the Royal College of General Practitioners of the UK National Conference that was funded by Novo Nordisk. PA and CA did half a day's consultancy for Weight Watchers. None of these activities resulted in personal payments.
